# IGF1 receptor-targeted black TiO_2_ nanoprobes for MRI-guided synergetic photothermal-chemotherapy in drug resistant pancreatic tumor

**DOI:** 10.1186/s12951-022-01525-3

**Published:** 2022-07-06

**Authors:** Kaiwei Xu, Lufei Jin, Liu Xu, Yuchao Zhu, Lu Hong, Chunshu Pan, Yanying Li, Junlie Yao, Ruifen Zou, Weiwei Tang, Jianhua Wang, Aiguo Wu, Wenzhi Ren

**Affiliations:** 1 Department of Radiology, the Affiliated Hospital of Medical School, Ningbo University, 247 Renmin Road, Jiangbei District, Ningbo, 315020 Zhejiang China; 2 Cixi Institute of Biomedical Engineering, International Cooperation Base of Biomedical Materials Technology and Application, Chinese Academy of Science (CAS) Key Laboratory of Magnetic Materials and Devices & Zhejiang Engineering Research Center for Biomedical Materials, Ningbo Institute of Materials Technology and Engineering, CAS, 1219 ZhongGuan West Road, Ningbo, 315201 China; 3 Advanced Energy Science and Technology Guangdong Laboratory, Huizhou, 516000 China; 4 Key Laboratory of Diagnosis and Treatment of Digestive System Tumors of Zhejiang Province, Ningbo, 315016 China

**Keywords:** Pancreatic tumor, Drug resistance, Targeted photothermal therapy, Black TiO_2_ nanoparticles

## Abstract

Pancreatic ductal adenocarcinoma (PDAC) is among the deadliest malignant tumors with features of matrix barrier caused poor drug permeability, and susceptibility to drug resistance. Herein, a PDAC and its stromal cell dual-targeted photothermal-chemotherapy strategy is explored to loosen the matrix and reverse drug resistance. To achieve this goal, black TiO_2_-Gd nanocomposites were conjugated with insulin like growth factor 1 (IGF1), and loaded with gemcitabine (GEM) to construct bTiO_2_-Gd-IGF1-GEM nanoprobes. In vitro results show that under 808 nm near-infrared irradiation, killing effect of the nanoprobes on drug-resistant MIA PaCa-2 cell is 3.3 times than that of GEM alone. In vivo experiments indicate the synergetic photothermal-chemotherapy not only loosens fibrous matrix of pancreatic tumor model, but also dramatically inhibits tumor growth, and almost completely eradicates the tumor after 12 days of treatment. In addition, relaxation rate of the nanoprobes is 8.2 times than commercial contrast agent Magnevist, therefore boosts the signal of magnetic resonance imaging in pancreatic tumor. In conclusion, our results reinforce that the prepared nanoprobes are promising to break matrix barrier and overcome drug resistance in PDAC.

**Supplementary Information:**

The online version contains supplementary material available at 10.1186/s12951-022-01525-3.

## Introduction

Pancreatic ductal adenocarcinoma (PDAC) is a severely malignant tumor, with a poor 5-year survival rate of only 9%, and it is estimated to be the second largest contributor to cancer-related death after lung cancer by 2030 [1–3]. Chemotherapy, as a major treatment method for advanced PDAC patients, is commonly unsatisfactory for PDAC, and the effective rate of first line drug gemcitabine (GEM) is less than 20% [4, 5]. There are many causes for chemotherapy failure in PDAC, and two factors are primary. First, PDAC is coated by a “scar”-like matrix barrier, which consists of pancreatic stellate cells, fibroblasts, infiltrating inflammatory cells, and collagen fibers. This dense anatomic structure increases intratumor pressure and decreases blood supply in PDAC, consequently weakens drug delivery capability into tumor. Second, PDAC cell is usually susceptibility to drug resistance [6–8]. Therefore, how to break the matrix barrier, improve drug delivery, and reverse drug resistance are main challenges in current stage of PDAC chemotherapy.

In recent years, with the rapid development of nanotechnology, various nanoprobes with unique properties have provided new opportunities for tumor diagnosis and treatment [9–15]. To break through the dense fibrous stroma of pancreatic cancer, plenty of studies have been explored with different strategies, such as depleting the glycans in matrix membrane, reducing the production of stromal cells via inhibition of signal pathway, and fibrous matrix loosen through photothermal nanoprobes [16–18]. When it comes to drug resistance, there are complex mechanisms related, such as aberrant gene expression, deregulated signal pathways, and regulated rate-limiting enzymes, while nanoprobes also exhibit promising in drug-resistance overcoming [19–21]. Photothermal nanoparticles as drug carriers, such as gold nanoshells, gold nanorods, WO_3_ and WS_2_, have been reported to destroy the dense matrix barrier of pancreatic cancer and enhance the chemosensitivity of drug-resistant cells, and therefore can eradicate drug-resistant PDAC cells [22–26]. These studies prove nanoprobes exert a great killing effect on PDAC when combined with photothermal-chemotherapy synergistic therapy, which is a promising tumor treatment method.

Given all of that, nanoprobe-based photothermal-chemotherapy strategy has emerged potential application in treatment of PDAC through hyperthermal induced matrix loosening, drug penetration improving, as well as drug resistance overcoming. Although targeting molecule-modified nanoprobes have been convinced to raise drugs accumulation in tumor. However, most of actively targeted nanoprobes are only designed to bind with PDAC cells, while are lack of targeting on both of cancer cells and stromal cells which probable more conducive for drug penetration and accumulation in PDAC. It is obviously that select an optimum binding site is the primary factor for dual targeted drug delivery. Insulin like growth factor 1 receptor (IGF1R) is ubiquitously expressed in 40–90% of PDAC cells and stromal cells, but is lowly expressed in normal pancreas. More excitingly, the expression of IGF1R is further elevated in drug-resistant PDAC cells [6, 27]. Meanwhile, studies have found that IGF1R plays critical functions in occurrence, development, invasion and metastasis of PDAC mainly through K-Ras or MAPK mediated signal pathways. Therefore, IGF1R is an ideal binding site for design of dual targeted drug delivery system.

**Fig. 1:**
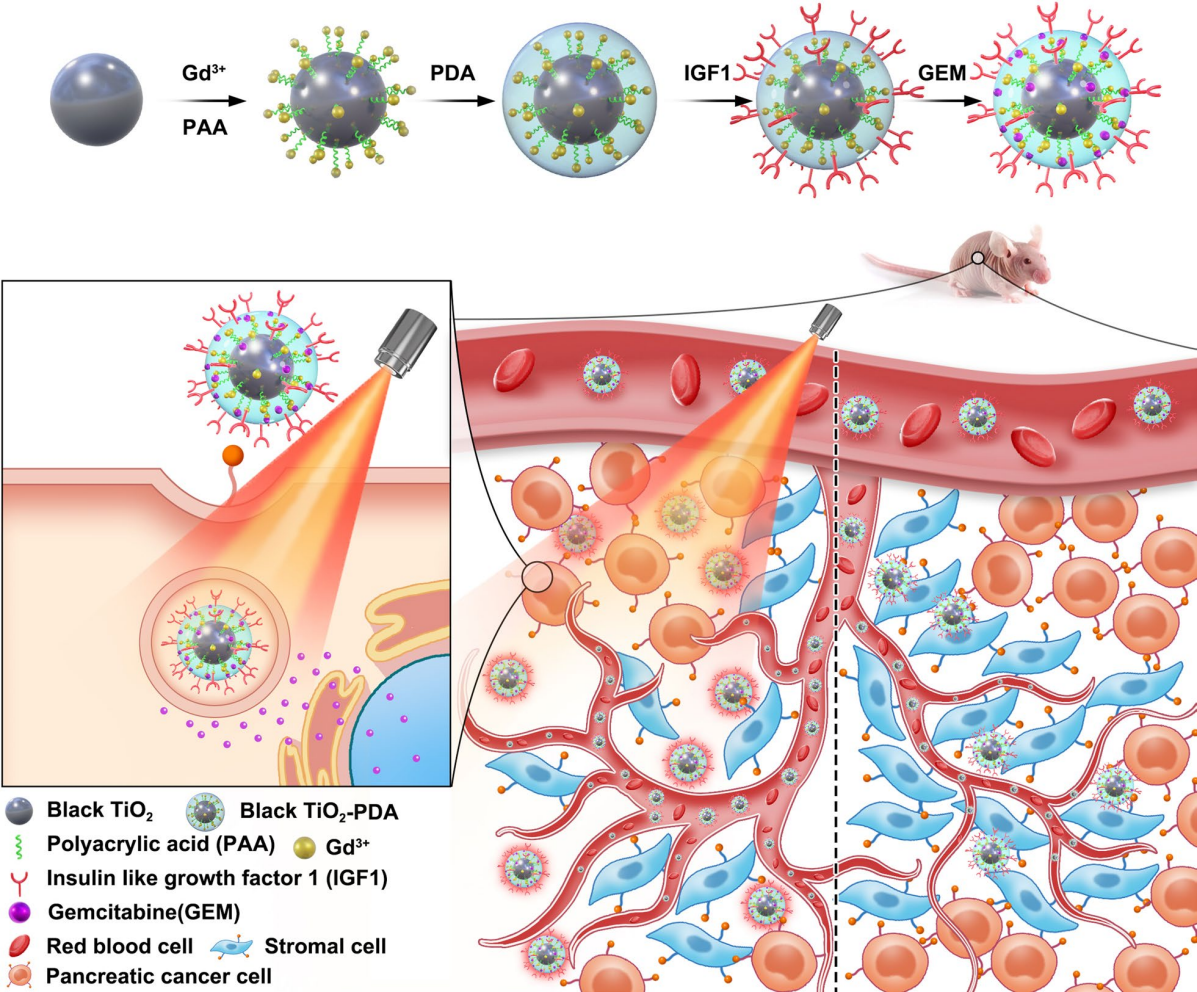
Illustration of GEM loaded, IGF1 conjugated, black TiO_2_-based nanoprobes for 808 nm NIR triggered synergistic photothermal-chemotherapy in drug-resistant pancreatic cancer

Besides target molecule, photothermal agent is another essential factor in photothermal-chemotherapy strategy. Black TiO_2_ (bTiO_2_) nanoparticles, with the advantages of elevated photothermal conversion efficiency, stable structure, and low toxicity, have been initially applied in photothermal therapy by our group [28]. Soon afterwards, bTiO_2_ nanoparticles were widely explored in cancer treatments, such as doxorubicin carriers in photothermal-chemotherapy of breast cancer, modified with CD133 antibody and DOTA-Gd for MRI-guided photothermal therapy of pancreatic cancer stem cells, as well as other reported bTiO_2_-based photothermal-photodynamic therapy, photothermal-sonodynamic therapy and so on [29–33]. All these findings prove bTiO_2_ are promising nanoprobes in photothermal-chemotherapy of cancer. Herein, to break the matrix barrier and reverse drug resistance, bTiO_2_ nanoparticles were coupled with gadolinium acetate, IGF1 polypeptide, and GEM to construct bTiO_2_-Gd-IGF1-GEM nanoprobes. As shown in Fig. 1, the bTiO_2_-Gd-IGF1-GEM nanoprobes are designed to specially bind with both of PDAC cells and stromal cells, subsequently loosen the matrix barrier, enhance drug permeability and reverse drug-resistance under NIR triggered photothermal effect. In vivo results show that after 12 days of treatment, average tumor size is 0.3 ± 0.4 mm^3^ in photothermal-chemotherapy group, while the size is 609.0 ± 153.7 mm^3^ in control group, which well clarifies the promising application prospect of above proposed nanoprobes in PDAC.

## Materials and methods

### Reagents

Dopamine hydrochloride, sodium borohydride (NaBH_4_), TiO_2_ nanoparticles, polyacrylic acid MW2000 (PAA MW2000), tris (hydroxymethyl) aminomethane acetate salt (C_4_H_11_NO_3_·C_2_H_4_O_2_), 3-(4,5-di-methylthiazol-2-yl)-2,5-diphenyltetrazolium bromide (MTT), dimethyl sulfoxide (DMSO) were acquired from Aladdin Industrial Inc (Shanghai, China). 1-Ethyl-3-[3-dimethylaminopropyl] carbodiimide hydrochloride (EDC), Gadolinium nitrate hexahydrate (GdN_3_O_9_·6H_2_O), *N*-hydroxysuccinimide (NHS), Rhodamine 123 (C_21_H_17_C_l_N_2_O_3_) were obtained from Macklin Biochemical Co., Ltd (Shanghai, China). Carboxyl polyethylene glycol amino group (NH_2_-PEG-COOH) were obtained from Yare Bio Co., Ltd (Shanghai, China). Recombinant human IGF1 protein (ab270062) was purchased from Abcam Bio Co., Ltd (England). Gemcitabine (C_9_H_11_F_2_N_3_O_4_) was purchased from MedChemExpress Co., Ltd (USA). DMEM medium, 0.25% Trypsin–EDTA was acquired from Gibco (Grand Island, USA). Penicillin–streptomycin liquid, phosphate buffer saline (PBS) came from GE Healthcare HyClone (LA, USA). 4% formaldehyde solution, Phalloidin, Fluorescein Isothiocyanate Labeled (FITC), Hoechst 33258 (C25H24N6O·3HCl), RIPA buffer were obtained from Solarbio Biotech Co., Ltd (Beijing, China). The calcein-AM/PI double stain kit came from Yeasen Biotechnology Co., Ltd (Shanghai, China). Protease inhibitor Cocktail was purchased from Bimake Biotechnology Co., Ltd (USA). ECL chromogenic solution was purchased from Adavansta Co., Ltd (USA). PVDF membrane was purchased from Millipore Co., Ltd (USA). Skim milk powder was purchased from Yili Co., Ltd (Inner Mongolia, China). Pre-stained protein MARKER (BIO-RAD) was purchased from Thermo Fisher Scientific Inc (USA). BCA Protein Assay Kit was obtained from Beyotime Biotech Co., Ltd (Shanghai, China). β-Actin Mouse Monoclonal Antibody was purchased from Santa Cruz Biotech Co., Ltd (USA). Anti-IGF1R antibody was acquired from Bioss Biotech Co., Ltd (Beijing, China).

### Preparation of bTiO_2_ nanoparticles

Using NaBH_4_ as the reducing agent, the TiO_2_ was reduced to bTiO_2_. First, 1 g of TiO_2_ powers were combined with NaBH_4_ at a mass ratio of 1:1 for 30 min, then were put inside a tube furnace with a heating rate of 10 °C/min. The temperature was increased to 350 °C in an argon atmosphere, and the reaction lasts for 3 h. The prepared bTiO_2_ nanoparticles were rinsed with ultrapure water and were centrifuged thrice minimum to remove NaBH_4_. Finally, the bTiO_2_ nanoparticles were dried for later using.

### Synthesis of bTiO_2_-Gd-COOH

Gadolinium acetate tetrahydrate, dopamine hydrochloride and NH_2_-PEG-COOH were employed for the modification of the bTiO_2_ nanoparticles. 100 mg of bTiO_2_ powders were dissolved into ultrapure water (100 mL) and were dispersed via ultrasound. Then ultrapure water was introduced to the bTiO_2_ solution to 90 mL. 500 mg of PAA2000 powders were dissolved into 10 mL of ultrapure water and were stirred magnetically. Next, the bTiO_2_ mixture was combined with PAA2000 and stirred for 5 h at room temperature (RT). The dispersions were next collected and centrifuged at 12,000 rpm for thrice. The bTiO_2_-PAA nanoparticles were dissolved in 90 mL of ultrapure water. 10 mL of Gadolinium Acetate Tetrahydrate (1 mg mL^−1^) were added in bTiO_2_-PAA dispersions. After stirring for 3 h, the dispersions were collected and centrifuged at 12 000 rpm thrice, then obtained bTiO_2_-Gd were dispersed in 10 mL of ultrapure water. The bTiO_2_-Gd dispersions were added in 80 mL of 10 mM Tris (PH8.5). After ultrasound for 10 min, the 2 mL of dopamine hydrochloride (75 mg mL^−1^) was introduced dropwise into this reaction system and stirring at RT for 1 h. The bTiO_2_-Gd-PDA dispersions were collected by centrifugation. Subsequently, the dispersions were dispersed into 50 mL of ultrapure water. After ultrasound for 10 min, NH_2_-PEG-COOH solutions were added dropwise to the bTiO_2_-Gd-PDA and stirring for 24 h. Finally, the bTiO_2_-Gd-COOH dispersions were centrifuged at 12,000 rpm, prior to storage in 10 mL of ultrapure water.

### Conjugation of IGF1 Peptide

bTiO_2_-Gd-COOH nanoparticles were conjugated with IGF1 peptide by EDC/NHS activated amide bonds. Briefly, 5 mL of bTiO_2_-Gd-COOH dispersions were resuspended in 45 mL of ultrapure water. 15 mg EDC and 20 mg NHS were dissolved in the dispersions, followed by stirring at RT for 20 min. Subsequently, 100 μL of IGF1 peptide (1 μg μL^−1^) were added and stirred for 12 h. The prepared bTiO_2_-Gd-IGF1 was collected by centrifugation and stored at 4 degrees.

### Preparation of bTiO_2_-Gd-GEM and bTiO_2_-Gd-IGF1-GEM

2 mL of GEM (4 mg mL^−1^) were introduced to 8 mL of bTiO_2_-Gd-COOH (0.75 mg mL^−1^) and bTiO_2_-Gd-IGF1 (0.75 mg mL^−1^) respectively, followed by stirring for 24 h. GEM loaded nanocarriers (bTiO_2_-Gd-GEM or bTiO_2_-Gd-IGF1-GEM), the nanoparticles were centrifuged, followed by storage in ultrapure water. The GEM loading efficiency was computed based on the drug’s UV–visible absorption, as we reported earlier [34, 35]. In short, following centrifugation, supernatants with GEM were obtained. Next, we assessed the accumulated and free GEM (0–100 μg mL^−1^) UV–visible absorptions, as well as the GEM concentration–absorption curve. Lastly, the GEM loading amount on the nanocarriers was computed as follows: loading efficiency = ((total GEM − GEM in supernatant) ÷ nanocarrier amount) × 100%.

### Characterization

The nanoprobes microstructure was evaluated via transmission electron microscopy (FEI Tecnai F20). The nanoprobe zeta potential and nanoprobe size distribution were next examined via a particle-size zeta potential analyzer (Nano ZS, Malvern Instruments Ltd, England), and by implementing a UV–visible spectrophotometer (T10CS, Persee General Equipment Co., Ltd, China), UV–visible absorption spectra were assessed. Infrared (IR) spectra of the nanoprobes were obtained from a Fourier transform infrared spectrometer (FTIR, Thermo Nicolet 6700, US). The nanoprobe element content was measured via induction coupled plasma optical emission spectrometry (ICP-OES, Spectro Analytical Instruments GmbH, Germany). Further, by employing a MR scanner system utilizing a magnetic field of 1.5 T, MR imaging and relaxivity capabilities were tested. The nanoprobe photothermal stability and effectiveness were assessed via an infrared (IR) thermal imaging apparatus (Optris Infrared Thermometers, Germany) and 808 nm semiconductor laser (BWT Beijing LTD, China).

### Assessment of photothermal efficiency and photostability

To test photothermal properties, bTiO_2_-Gd-COOH nanoparticles with different Ti concentrations were added to cuvette and irradiated with various power densities from an 808 nm laser. The values of temperature for bTiO_2_-Gd-COOH dispersions were documented every 10 s using an IR thermal imaging apparatus (MAG-V30, Vst Light & Technology Ltd, China), and we simultaneously mapped the images of real-time thermal dispersion. We have summarized the different aforementioned situations below: (1) 1 mL of nanoparticles (150 μg mL^−1^ of Ti concentration) receiving irradiation with 808 nm laser with different power densities (0.4–2.0 W cm^−2^) for 10 min; (2) Varying nanoparticle concentrations (0–150 μg mL^−1^ of Ti concentration) were irradiated through an 808 nm laser (1.2 W cm^−2^) for 10 min; (3) For detecting nanoparticle photostability, the bTiO_2_-Gd-COOH dispersions (150 μg mL^−1^ of Ti concentration) were also irradiated with an 808 nm laser (1.2 W cm^−2^) every 5 min for over 6 on/off repeating cycles. The resulting images were collected, and the temperature values fitted in time–temperature curves.

### pH-responsive and NIR-triggered drug release

To evaluate drug release, in three dialysis bags, 2 mL bTiO_2_-Gd-IGF1-GEM (GEM: 3 μg mL^−1^) was placed, and each dialysis bag was immersed in 20 mL PBS buffer solution with pH of 7.4, 5.0 and 5.0 respectively. 1 mL of dialysate was withdrawn and the same quantity of PBS buffer was introduced to keep the same quantity of buffer equal at 1 h, 2 h, 3 h, 4 h, 5 h, 8 h, 12 h, 24 h, and 27 h, respectively. Upon collection of the solution, the solution with pH 5.0 received irradiation with a 1.2 W cm^−2^ near-infrared laser for 15 min. The drug release was monitored via UV–Vis spectrophotometer, and the drug release was computed based on the drug release dose absorption curve.

### In vitro MRI

The MRI and MR relaxivity of nanoprobes were evaluated by 1.5 T MRI scanner apparatus. The nanoprobes with varying Gd concentrations (0.025 mM, 0.05 mM, 0.1 mM, 0.2 mM, 0.4 mM) were placed in a tube to measure the relaxation rate. At the same time, the commercial contrast agent Magnevist was adjusted to the control Gd concentration. We obtained the specific longitudinal and transverse, namely (r_1_) and (r_2_) relaxivities from the rate of relaxation R1 (1/T_1_) and R2 (1/T_2_) slopes accordingly. The T_1_-weighted MRI characteristics of nanoparticles at varying concentrations of Gd were obtained from the same MRI apparatus, and parameters were adjusted as shown below: T_1_ spin echo sequence, TE = 18.20 ms, TR = 200.00 ms, FOV = 100 mm × 100 mm, slice width = 2.0 mm, 1.5 T.

### Expression of IGF1R in different PDAC cell lines

The IGF1R levels in the hTERT-HPNE pancreatic ductal epithelial cells, BxPC-3 PDAC cells, Panc-2 PDAC cells, and MIA PaCa-2 PDAC cells were quantified via western blot analysis. Firstly, the total proteins of the above four cells were extracted and the protein quantification and concentration were measured. Then, 10% concentration of concentrated gel and separation gel were selected according to the molecular weight of the protein. The solidified PAGE gel was installed on the electrophoresis tank, 1× electrophoresis buffer between two pieces of glass to join until more than plastic plane, and gently pull out a comb. According to different protein concentrations, protein samples of equal mass were taken and added to each well. Meanwhile, appropriate protein markers were selected and added to the outermost well according to different protein molecular weights. Constant pressure method was used for protein electrophoresis. PVDF membrane of appropriate size was selected for membrane transfer. After membrane transfer, PVDF membrane was carefully clamped out and sealed with protein. The PVDF membrane was clamped out and cleaned repeatedly for 3 times. The PVDF membrane was treated with the configured primary antibody, and the membrane was kept on a horizontal shaker at 4 °C for 6 h. Finally, primary antibody was recovered, PVDF membrane was clipped out and washed 3 times, PVDF membrane was incubated with the configured secondary antibody, and placed on a horizontal shaker at 4 °C for 1 h. Upon incubation, PVDF membrane was clipped out and washed 3 times. Then, appropriate ECL chemiluminescence was configured according to the size of the membrane and added to the PVDF membrane. The membrane was exposed in a chemiluminescence imager, and the results were recorded.

### Cell culture

The MIA PaCa-2 PDAC, Panc-2 PDAC, BxPC-3 PDAC and hTERT-HPNE human pancreatic ductal epithelial cell lines were acquired from the Cell Bank of the China, Academy of Sciences (Shanghai, China). The MIA PaCa-2, Panc-2, BxPC-3 and hTERT-HPNE cell lines were grown in complete DMEM medium, containing 10% FBS, 100 U mL^−1^ penicillin and 100 U mL^−1^ streptomycin, and incubated at 37 °C with 5% CO_2_.

### Cytotoxicity of nanoprobes

For cytotoxicity analysis of bTiO_2_-Gd-COOH and bTiO_2_-Gd-IGF1, MIA PaCa-2 PDAC cells were seeded in 96-well plates (1 × 10^4^ cells per well) and grown for 24 h. Next, different concentrations of nanoprobes (50–300 μg mL^−1^) were added into each well. Following 20 h, 10 μL of MTT reagent (5 mg mL^−1^ in PBS) was introduced to each well and kept for additional 4 h. Finally, the mediums were suctioned off, and formazan crystals were resuspended by DMSO. The absorbances of formazan solutions were detected using a microplate absorbance reader (BioradiMARK™, USA) at 490 nm and 550 nm, and cell viabilities were computed.

### Cellular uptake characterization

To measure cell-targeting capability of IGF1 conjugated nanoprobes, X-ray fluorescence microscopy at Shanghai Synchrotron Radiation Facility (SSRF, Shanghai, China) was used. First, cells were seeded on sterile Malay films and placed in a cell incubator for 24 h, then cultured in DMEM and bTiO_2_-Gd-COOH, bTiO_2_-Gd-IGF1, bTiO_2_-Gd-IGF1-GEM for 2 h, respectively. Next, cells were twice PBS-rinsed before fixation in 4% formaldehyde solution. Finally, elemental fluorescence of Ti and Cl in cells was obtained. The X-ray energy was 10 keV, and the beam spot was 0.5 × 0.5 μm^2^. Elemental maps of Ti, Cl in cells were acquire.

For statistical analysis of targeting ability, MIA PaCa-2 cells were seeded in 6-well plates for 24 h, and maintained with bTiO_2_-Gd-COOH, bTiO_2_-Gd-IGF1 for another 2 h. Next, the cells were digested by nitric acid and hydrofluoric acid. The element content in cells were determined via ICP-OES.

For furthermore proving targeting capability, bTiO_2_-Gd-COOH and bTiO_2_-Gd-IGF1 nanoprobes were labelled by fluorescence dye rhodamine 123, and were determined by laser confocal microscope at excitation wavelength of 488–505 nm and emission wavelength of 515–575 nm. MIA PaCa-2 cells (1 × 10^5^ cells) were grown on a 35 mm petri dish and placed in an incubator for 24 h, prior to treatment with the same concentration of DMEM, bTiO_2_-Gd-COOH, and bTiO_2_-Gd-IGF1 for 2 h. Next, the cells were PBS-rinsed before fixation in 4% paraformaldehyde, and treatment with 0.2% Triton X-100. Followed, 1% BSA solution was used for blocking non-specific interacting sites, and the cells were incubated by employing 50 μg mL^−1^ FITC-Ghost Pencyclic peptide and 2 μg mL^−1^ Hoechst 33342 for cytoskeleton and nucleus staining.

### Photothermal therapy synergistic chemotherapy evaluation

MIA PaCa-2 cells were grown in the plates containing 96 wells (1 × 10^4^ cells per well), followed by incubation in DMEM, GEM, bTiO_2_-Gd-COOH, bTiO_2_-Gd-GEM, and bTiO_2_-Gd-IGF1-GEM or bTiO_2_-Gd-IGF1 for 2 h. Ti concentrations of bTiO_2_ contained groups were 150 μg mL^−1^, the GEM concentration of the GEM alone group was consistent with that of the other groups, which was 3 μg mL^−1^. 2 h later, the old media was suctioned off and fresh DMEM was added, and then each well underwent irradiation with 808 nm NIR laser for 1–4 min at power densities of 1.2 W cm^−2^, respectively. MTT was subsequently employed to assess cell viability.

### In vivo toxicity evaluation of nanoprobes

For animal experiments, animal care and handling procedures were in agreement with the guidelines of the Regional Ethics Committee for Animal Experiments at Ningbo University (Permit No. SYXK Zhe 2019-0005). To assess nanoparticle toxicity in vivo, 12 normal Balb/c mice (4–6 weeks old) were arbitrarily separated into 3 groups. The mice received intravenous injection of 150 μL of PBS, bTiO_2_-Gd-COOH or bTiO_2_-Gd-IGF1. The nanoprobe dosage was 20 mg kg^−1^. This treatment was carried on for a month, and we documented their weight and behavior. At the end of the month, all mice were euthanized. One mouse from each group was tested for routine blood. Major organs like heart, liver, spleen, lung and kidney were fixed in 10% formalin prior to hematoxylin and eosin (H&E) staining.

### In vivo imaging and synergetic photothermal-chemotherapy

Tumor models were developed in male balb/c nude mice (4–6 weeks). MIA PaCa-2 cells (1 × 10^6^ cells for each mouse) in DMEM were subcutaneously injected in the right flank of the animals. The mice were arbitrarily separated into 6 groups (5 mice per group). The tumor size was measured with a digital caliper. Tumor volume = 4/3 × π × (tumor radius)^3^. Our experiments were initiated once tumors reached 50 mm^3^. The mice received intratumoral administration of 100 μL of PBS, GEM, bTiO_2_-Gd-IGF1, bTiO_2_-Gd-IGF1-GEM. The concentration of GEM was 3 μg mL^−1^ in GEM groups and the concentration of Ti concentration was 150 μg mL^−1^ in the bTiO_2_ groups. Meanwhile, for MR imaging, commercial contrast agent magnevist with a Gd concentration of 3 μg mL^−1^ was injected. Following 24 h of incubation, mice underwent intraperitoneal injection of 8 wt% of chloral hydrate solution, followed by irradiation, in the presence or absence of 808 nm NIR laser for 5 min at 1.2 W cm^−2^. The tumor temperature alterations were documented using an infrared thermometer (PI400, Optris, Germany). From each group, one mouse was arbitrarily chosen and euthanized following the irradiation of NIR, and the tumor injury was analyzed via H&E staining. We monitored tumor sizes for 12 days and computed the volumes based on the aforementioned tumor volume formula. Mice body weights were also documented for 2 weeks.

### Statistical analysis

Data are expressed as mean ± standard deviation, and via the Student’s t test were compared. P < 0.05 was adjusted as the significance threshold.

## Results and discussion

### Characterizing the bTiO_2_-Gd-IGF1-GEM nanoprobes

The bTiO_2_-Gd-IGF1-GEM synthesis process is showed in Fig. 1. To improve dispersibility of bTiO_2_, meanwhile to supply coordination sites for contrast agent Gd, bTiO_2_ nanoparticles were coated with PAA through hydrogen bonding and charge adsorption between positive charge of bTiO_2_ and carboxyl of PAA. Then the nanoparticles were complexed with Gd^3+^ through coordination bonds to form bTiO_2_-Gd nanoprobes. Polydopamine (PDA), with phenolic hydroxyl polymerized special cyclic structures, can supply binding sites for peptide conjugation and drug loading, and is usually used for surface coating of nanoparticles [36, 37]. To further improve dispersibility of obtained bTiO_2_-Gd-PDA, the nanoprobes were conjugated with amino group of NH_2_-PEG-COOH through Michael addition reaction. The formed bTiO_2_-Gd-COOH nanoprobes have good dispersibility. Gemcitabine (GEM), a first-line chemotherapy drug for PDAC, was loaded by bTiO_2_-Gd-COOH via pai–pai stacking and/or hydrogen binding [38]. Finally, IGF1 peptides underwent conjugation on the nanoparticles via EDC/NHS-activated amide bond to form bTiO_2_-Gd-IGF1 nanoprobes [21].

**Fig. 2:**
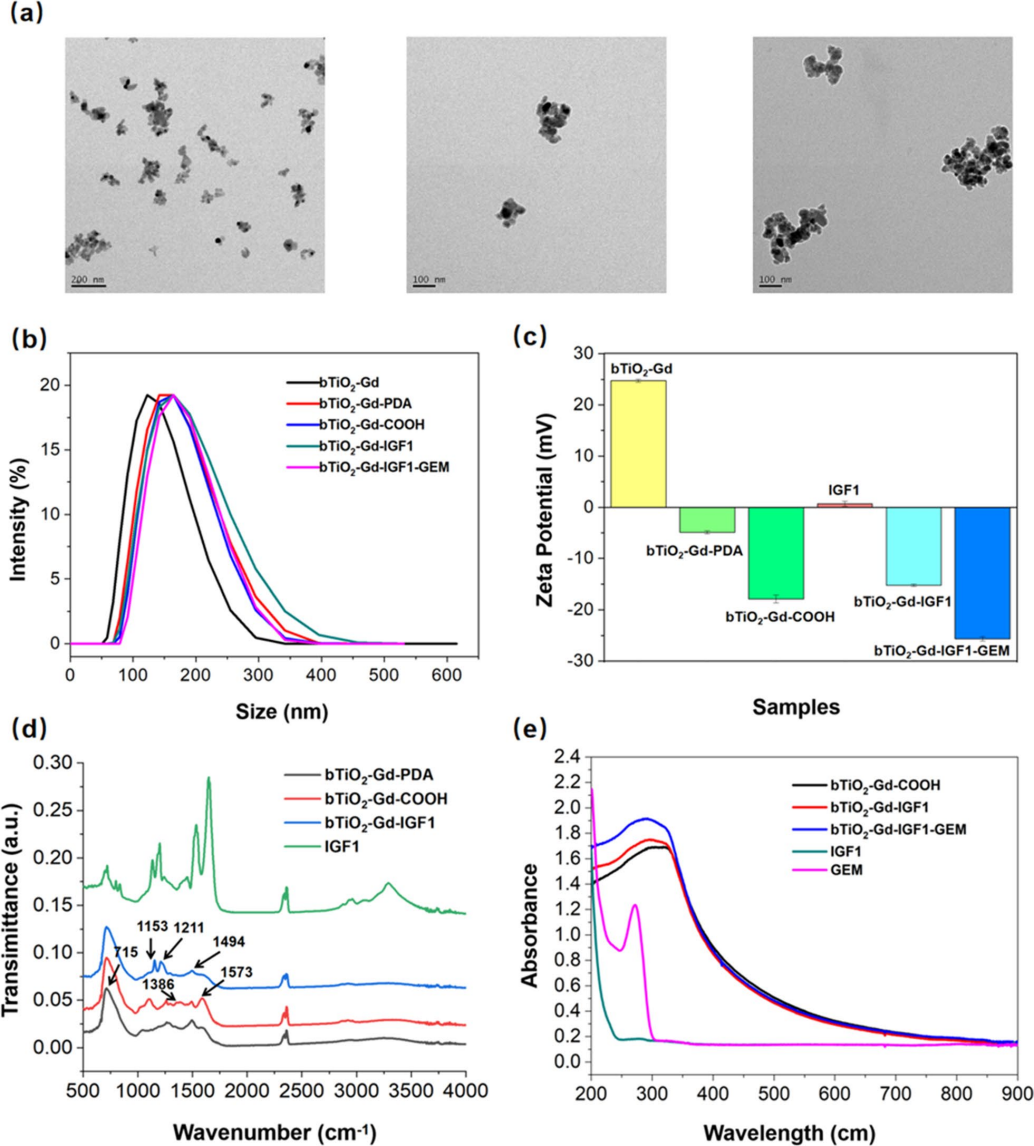
Characterization of physical and chemical properties of bTiO_2_-Gd-IGF1-GEM nanoprobes. **a** TEM images of bTiO_2_-Gd, bTiO_2_-Gd-PDA and bTiO_2_-Gd-IGF1. **b**–**e** Particle sizes, zeta potentials, FTIR spectra and UV–Vis absorption spectra of the nanoprobes

As shown in Fig. 2a, TEM images indicate diameters of bTiO_2_-Gd, bTiO_2_-Gd-PDA and bTiO_2_-Gd-IGF1 nanoprobes are 60 nm, from left to right respectively. The mean hydrated size of bTiO_2_-Gd, bTiO_2_-Gd-PDA, bTiO_2_-Gd-COOH, bTiO_2_-Gd-IGF1, bTiO_2_-Gd-IGF1-GEM are about 123.3 nm, 153.2 nm, 149.2 nm, 158.8 nm and 159.9 nm, respectively, in Fig. 2b. The data show that with modification of related components, particle sizes of the nanoprobes gradually increase, bTiO_2_-Gd-IGF1-GEM of this size has good biocompatibility and is still suitable for tumor treatment. In addition, zeta potentials of bTiO_2_-Gd, bTiO_2_-Gd-PDA, bTiO_2_-Gd-COOH, IGF1 polypeptide, bTiO_2_-Gd-IGF1 and bTiO_2_-Gd-IGF1-GEM are about 24.73 mV, − 4.85 mV, − 17.87 mV, 0.67 mV, − 15.27 mV and − 25.67 mV, respectively. In general, traditional white TiO_2_ nanoparticles show negative zeta potential owing to surface hydroxyl groups. Our previous investigation prove bTiO_2_ nanoparticles exhibit positive zeta potential which probably due to reduction induced abundant oxygen vacancies on the nanoparticles surface [20]. Besides, coordinated with Gd^3+^ also increase positive charge, so the surface charge of bTiO_2_-Gd nanoprobes appear positive charge. After coating with PDA, surface charge of bTiO_2_-Gd-PDA nanoprobes are changed to negative. Zeta potential of bTiO_2_-Gd-IGF1-GEM reaches − 25.67 mV after modification with NH_2_-PEG-COOH, IGF1 and GEM, which is more suitable for in vivo delivery when compared with positive surface charge.

Figure 2d is the FTIR spectra of nanoprobes and IGF1 polypeptides. There is a sharp peak near 715 cm^−1^ in bTiO_2_-Gd-PDA, bTiO_2_-Gd-COOH, and bTiO_2_-Gd-IGF1, which is attributed to characteristic absorption peak of Ti–O [39]. After modified NH_2_-PEG-COOH through Michael addition reaction, bTiO_2_-Gd-COOH nanoprobes exhibit carboxylic acid symmetrical and asymmetrical stretching vibrations at 1386 cm^−1^ and 1573 cm^−1^ [40]. In bTiO_2_-Gd-IGF1 nanoprobes, there are characteristic peaks at 1494 cm^−1^, 1153 cm^−1^, and 1211 cm^−1^, which suggests IGF1 polypeptide has been successful connected with bTiO_2_-Gd-COOH nanoprobes. UV–visible spectra of various samples are provided in Fig. 2e, relative to bTiO_2_-Gd-COOH and bTiO_2_-Gd-IGF1, the absorption peak of the bTiO_2_-Gd-IGF1-GEM nanoprobes exhibit slightly red-shift which attributing to gemcitabine loading.

### MRI and photothermal performance properties

**Fig. 3:**
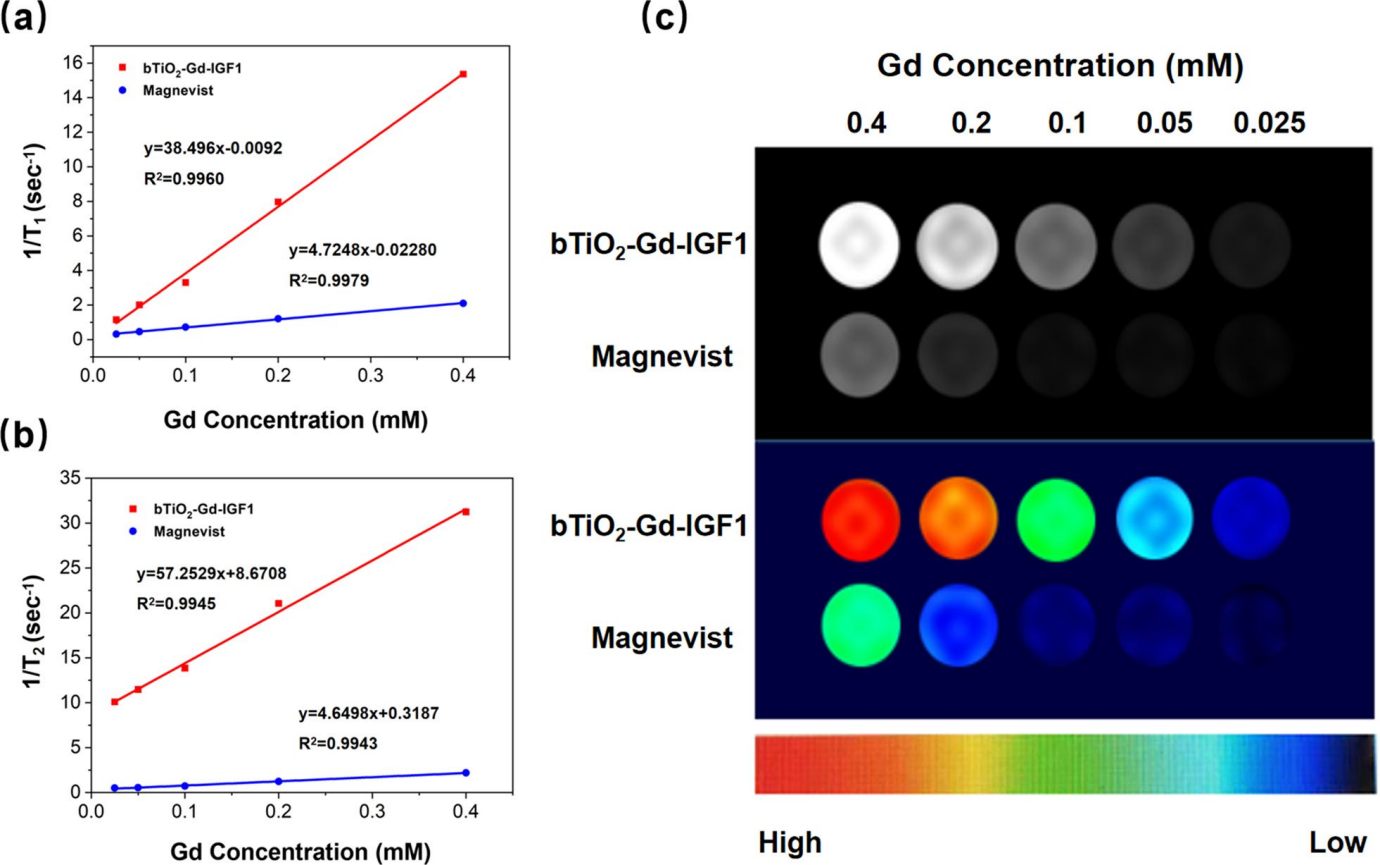
MR characteristics of the bTiO_2_-Gd-IGF1 nanoprobes. The fitted longitudinal (**a**) and transverse (**b**) relaxation (1/T_1_ and 1/T_2_) plots of bTiO_2_-Gd-IGF1 and Magnevist at the similar concentrations of Gd. **c** T_1_-weighted imaging of bTiO_2_-Gd-IGF1 nanoprobes and Magnevistat various Gd concentrations, and their pseudo-color photos

MR imaging and relaxivities of the nanoprobes were evaluated by 1.5 T MRI scanner system. Commercial contrast agent Magnevist is employed as the control. As depicted in Fig. 3a, r_1_ value of bTiO_2_-Gd-IGF1 is 38.496 mM^−1^ s^−1^, while the value of Magnevist is 4.7248 mM^−1^ s^−1^ at the similar concentration of Gd. It should be pointed out that the bTiO_2_-Gd-IGF1 r_1_ value is approximately 8.2 folds greater than the Magnevist. The markedly high bTiO_2_-Gd-IGF1 r_1_ value is likely because of gadolinium coordinated by PAA with hydrophilic high molecular weight, which can enhance the rotational correlation time (τR) [19]. Similarly, Fig. 3b shows r_2_ values of bTiO_2_-Gd-IGF1 is also markedly elevated (r_2_ = 57.2529 mM^−1^ s^−1^), compared to Magnevist (r_2_ = 4.6498 mM^−1^ s^−1^). The bTiO_2_-Gd-IGF1 r_2_/r_1_ ratio is approximately 1.487, which illustrates the nanoprobes are good T_1_-weighted MRI contrast agent. Consequently, it is clearly indicated bTiO_2_-Gd-IGF1 nanoprobes exhibit brighter T_1_-weighted MRI signal than Magnevist at the same Gd concentration in Fig. 3c.

**Fig. 4:**
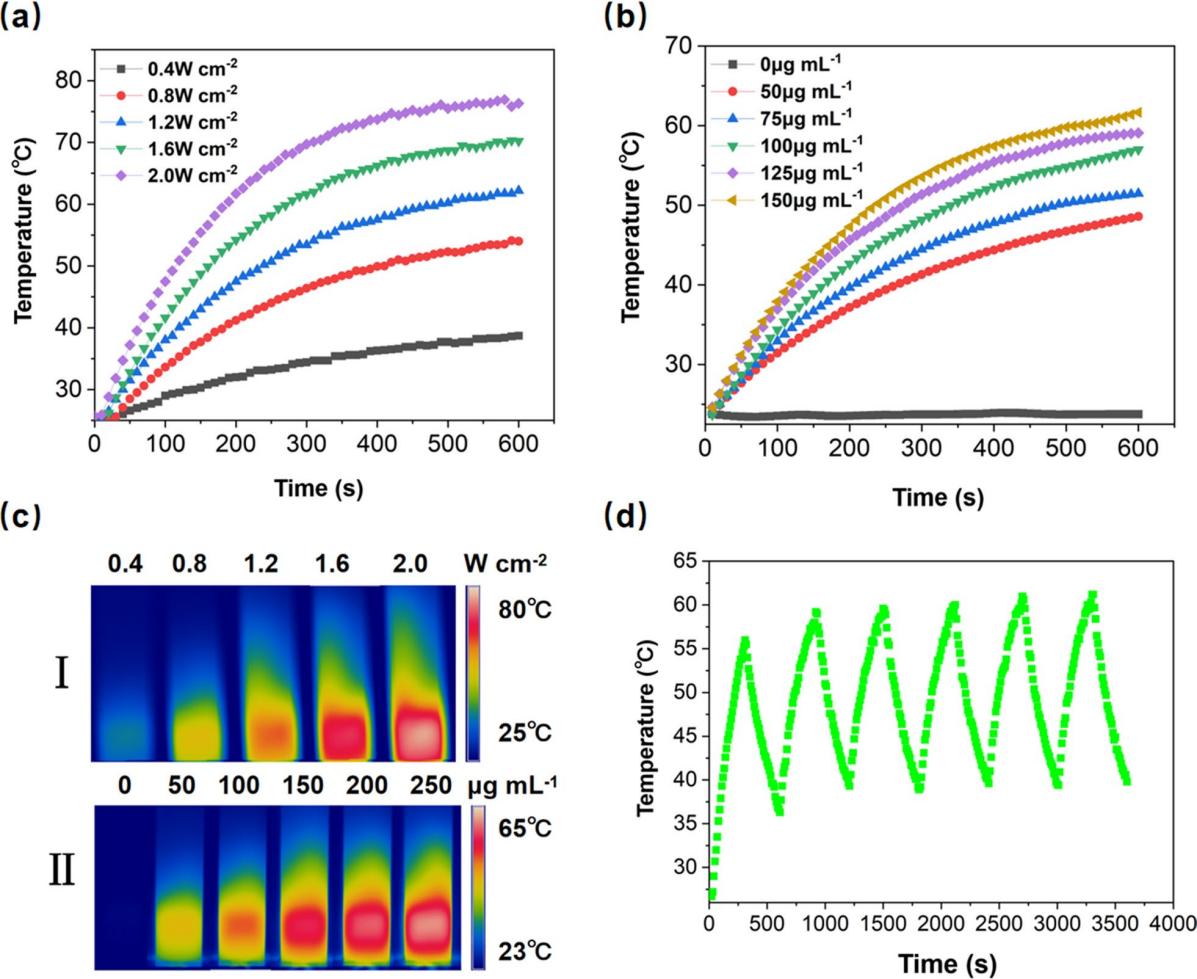
Photothermal properties of bTiO_2_-Gd-COOH. **a**, **c** I, bTiO_2_-Gd-COOH irradiated by 808 nm laser with power density of 0.4–2.0 W cm^−2^ for 10 min. **b**, **c** II, the nanoprobes at various concentrations irradiated with 1.2 W cm^−2^ 808 nm laser for 10 min. **d** Photothermal stability of bTiO_2_-Gd-COOH

Although previous reports have proved bTiO_2_ nanoparticles possess high photothermal capability [28, 29]. In this study, bTiO_2_ nanoparticles were modified by PAA, PDA, as well as NH_2_-PEG-COOH, therefore, photothermal performance of the prepared bTiO_2_-Gd-COOH nanoprobes were evaluated. Figure 4a shows 1 mL bTiO_2_-Gd-COOH dispersions (150 μg mL^−1^) were irradiated with 808 nm laser for 10 min. With 808 nm laser increase in power, the nanoprobe temperature rises quickly after 10 min. As expected, cancer cells can undergo rapid ablation following few minutes of exposure to 50 °C [41]. When the power density of 808 nm laser was 1.2 W cm^−2^, the temperature of bTiO_2_-Gd-COOH dispersions were 62.2 °C after 10 min irradiation, which is considered more suitable in followed experiment. Then, bTiO_2_-Gd-COOH nanoprobes underwent irradiation with 1.2 W cm^−2^ of NIR laser at various concentrations of Ti from 50 to 150 μg mL^−1^. As shown in Fig. 4b, 50 μg mL^−1^ of bTiO_2_-Gd-COOH nanoprobes reached 48.7 °C after irradiated by 808 nm laser for 10 min. In Fig. 4c, I and II are real-time photothermal images, which corresponds well with the curves of temperature in Fig. 4a, b, accordingly. In addition to photothermal performance, the photothermal stability of nanoprobes is also important. Figure 4d shows bTiO_2_-Gd-COOH nanoprobes were irradiated by an 808 nm laser at 1.2 W cm^−2^ for 6 cycles in 1 h. The temperature curve during heating and cooling is almost similar during in 6 cycles, which proves nanoprobes are stability during NIR irradiation.

### Drug release of nanoprobes

According to the above-mentioned formula, drug loading efficiency of bTiO_2_-Gd-IGF1-GEM is about 10.3%, which indicates high loading capacity of bTiO_2_-Gd-IGF1 nanoprobes. Additional file 1: Fig. S1 shows the drug release curves of bTiO_2_-Gd-IGF1-GEM at pH 7.4, pH 5.0, pH 5.0 + NIR, respectively. The cumulative release of GEM within 27 h was 2.7 times higher in the pH 5.0 group, compared to the pH 7.4 group. The weak acid promoted drug release is probably due to the protonation of amine groups on the PDA scaffolds and/or on the GEM molecules, which partially weeks the pai–pai interactions between PDA and drugs [38]. After near-infrared irradiation in pH 5.0 buffer, the total release of GEM further increased, reaching 40.8%, which is about 3.6 times than drug release at pH 7.4. NIR-responsive release pattern is mainly due to combined-photothermal effect can accelerate drug molecular motion and make GEM escape easily from the carriers [42, 43]. The above data indicate bTiO_2_-Gd-IGF1-GEM nanoprobe exhibit pH and NIR co-responded drug-release properties, consequently can enhance the concentration of GEM in the tumor's weakly acidic microenvironment.

### Screening of IGF1R expression in various PDAC cell lines

Western blot was employed to quantitatively determine the expression level of IGF1R in several PDAC cell lines, such as MIA PaCa-2 cells, hTERT-HPNE cells, BxPC-3 cells, and Panc-2 cells. Additional file 1: Fig. S2 shows that compared with hTERT-HPNE cells, BxPC-3 cells and Panc-2 cells, MIA PaCa-2 cells express the highest level of IGF1R. Furthermore, reports show that IGF1R express higher in drug-resistant pancreatic cells, and drug-resistance has been confirmed in MIA PaCa-2 cell line [27, 44]. Therefore, in the next experiment, we use MIA PaCa-2 cells for the cell experiment part of this experiment.

### Cell uptake of nanoprobes

**Fig. 5:**
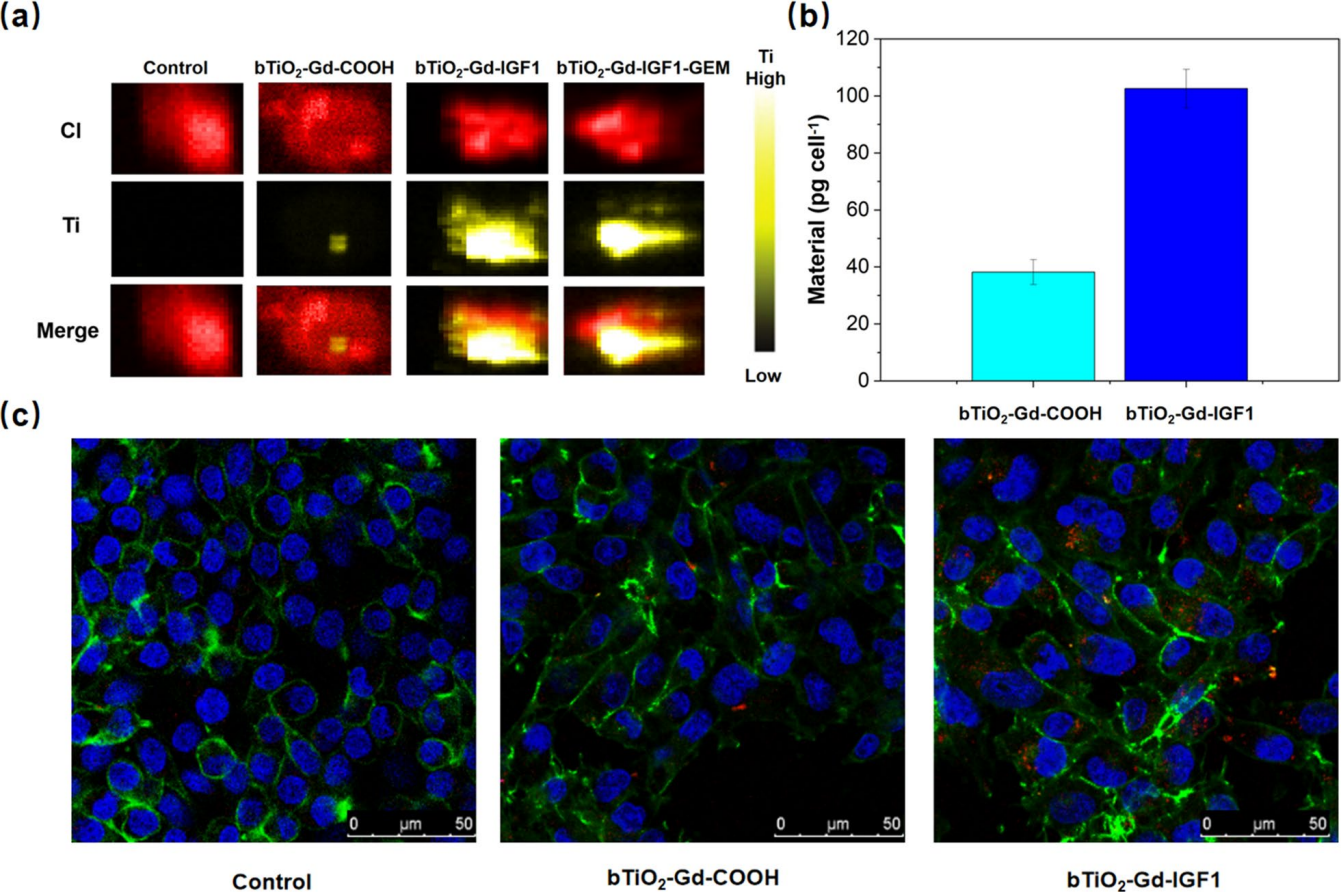
**a** XFM images of MIA PaCa-2 cells treated with bTiO_2_-Gd-COOH, bTiO_2_-Gd-IGF1 or bTiO_2_-Gd-IGF1-GEM. Biogenic chlorine element is depicted as red-bright, and Ti element of nanoprobes is depicted as yellow-bright. Scale bar is 10 μm. **b** ICP quantitative analysis, and **c** Confocal microscope images of nanoprobes incubated MIA PaCa-2 cells. Blue fluorescence is nucleus labeled with Hoechst 33342, green fluorescence is cell membrane stained with FITC-phalloidin, and red fluorescence is rhodamine stained nanoprobes. Scale bar is 50 μm

As described above, there are dense fibrous in pancreatic cancer. IGF1R is ubiquitously found in 40–90% of pancreatic cancer cells and stromal cells, which is ideal target sites for nanoprobes to break through the matrix and enter PDAC cells [6]. Here, in order to enhance IGF1R-target ability of nanoprobes, bTiO_2_-Gd-IGF1 nanoprobes were generated via conjugation of bTiO_2_-Gd-COOH with IGF1 peptides. The bTiO_2_-Gd-IGF1 nanoprobe targeting ability was evaluated by XFM, ICP-OES and laser confocal microscope. By taking advantage of XFM, direct visual and quantitative localization of the titanium element was acquired. As depicted in Fig. 5a, yellow fluorescence denotes the titanium element, and red fluorescence represents biogenic element chlorine, which maps cancer cells. Weak yellow fluorescence signals were observed in the bTiO_2_-Gd-COOH group, suggesting that MIA PaCa-2 cells have weak uptake of bTiO_2_-Gd-COOH nanoprobes. However, intensive yellow fluorescence appears in bTiO_2_-Gd-IGF1 and bTiO_2_-Gd-IGF1-GEM groups, which directly suggests that IGF1 peptide can enhance the targeting ability of nanoprobes. Followed, the target ability of nanoprobes on PDAC cells were quantitatively tested by ICP-OES. Figure 5b shows the amount of Ti element in the cells following treatment via bTiO_2_-Gd-COOH or bTiO_2_-Gd-IGF1 for 4 h. Similarly, the amount of Ti element of the cells in bTiO_2_-Gd-IGF1 group is significantly higher than bTiO_2_-Gd-COOH group, which indicates IGF1 peptide can improve the targeting ability of nanoprobes to MIA PaCa-2 cells. Finally, laser confocal microscope was employed for the observation of rhodamine 123 labeled nanoprobes uptake into MIA PaCa-2 cells. Figure 5c provides intracellular nanoprobe distribution in MIA PaCa-2 cells. Green fluorescence refers to the FITC-phalloidin labeled cell membrane, blue fluorescence shows Hoechst 33342 nuclear staining, and red fluorescence represents rhodamine 123 labeled nanoprobe. Compared with bTiO_2_-Gd-COOH group, bTiO_2_-Gd-IGF1 group exhibits more aggregation in the cells, which also indicates IGF1 peptide can improve targeting capability of the nanoprobes.

### Cytotoxicity assay

**Fig. 6:**
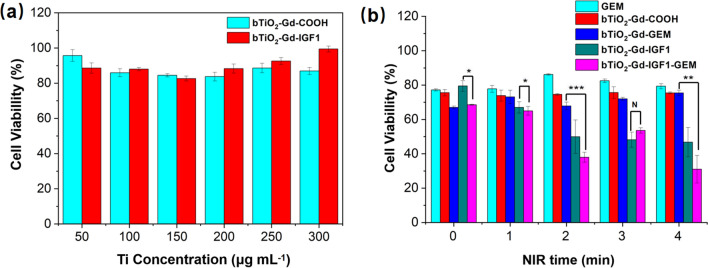
**a** Cytotoxicity of the nanoprobes in MIA PaCa-2 cells. **b** photothermal-chemotherapy of the nanoprobes in MIA PaCa-2 cells. Concentration of GEM is 3 μg mL^−1^, and the concentration of Ti is 150 μg mL^−1^. Data are presented as mean ± standard deviation (n = 3). ns > 0.05, *p < 0.05, **p < 0.01, ***p < 0.001 were evaluated via Student’s t test, and used as the significance threshold

Previous studies have confirmed bTiO_2_ nanoprobes are less toxic in vitro and in vivo [29, 30]. Here, we modified bTiO_2_ with Gd, dopamine, and IGF1 peptides, so it is still critical to assess nanoprobe toxicity. Figure 6a shows the cytotoxicity of bTiO_2_-Gd-COOH and bTiO_2_-Gd-IGF1 on MIA PaCa-2 cells. Following 24 h’s incubation, the cell viability is higher than 80% in the nanoprobes’ concentration range of 50–300 μg mL^−1^, which confirms the nanoprobes are low cytotoxicity. The cell survival rate of bTiO_2_-Gd-IGF1 incubated cells seem to increase when Ti concentration is over 200 μg mL^−1^. This may be attributed to more nanoprobes entered the cells and the cell color deepened, which affected the absorptions.

### PTT and chemotherapy of pancreatic cancer in vitro

Figure 6b shows PTT and chemotherapy of nanoprobes in PDAC cells. MIA PaCa-2 cells were maintained in DMEM, GEM, bTiO_2_-Gd-COOH, bTiO_2_-Gd-GEM, and bTiO_2_-Gd-IGF1 or bTiO_2_-Gd-IGF1-GEM for 2 h. When there is no laser irradiation, cell viabilities are 77.2 ± 0.6% (GEM), 75.5 ± 1.9% (bTiO_2_-Gd-COOH), 67.0 ± 0.8% (bTiO_2_-Gd-GEM), 79.5 ± 3.1% (bTiO_2_-Gd-IGF1) and 68.6 ± 0.3% (bTiO_2_-Gd-IGF1-GEM), respectively. The results show that only 22.8% of the cells can be killed by GEM after 2 h of incubation, which indicates drug-resistance of the cells. Cell survival rate in bTiO_2_-Gd-GEM and bTiO_2_-Gd-IGF1-GEM group is significantly lower than that in bTiO_2_-Gd-COOH and bTiO_2_-Gd-IGF1 group, which may be due to the killing effect of GEM. After NIR laser irradiation for 4 min, cell viabilities are 79.4 ± 1.5% (GEM), 75.4 ± 0.5% (bTiO_2_-Gd-COOH), 75.4 ± 1.6% (bTiO_2_-Gd-GEM), 46.8 ± 8.6% (bTiO_2_-Gd-IGF1) and 31.0 ± 8.6% (bTiO_2_-Gd-IGF1-GEM), respectively. Relative to PTT or chemotherapy alone, killing efficiency in photothermal-chemotherapy group is more significant. In particular, cell killing rate in bTiO_2_-Gd-IGF1-GEM group can reach 69%.

The in vitro photothermal-chemotherapy performance is also visually examined using live/dead cell staining via calcein AM/PI reagents. According to Additional file 1: Fig. S3, red fluorescence (PI) marks dead cells, and green flourescence (Calcein-AM) stains live cells. Most MIA PaCa-2 cells can survive under 1.2 W cm^−2^ of 808 nm NIR irradiation alone, indicating 808 nm NIR irradiation at 1.2 W cm^−2^ is safety to cells. However, MIA PaCa-2 cells are killed in varying degrees when the cells were maintained in the same concentrations of GEM, bTiO_2_-Gd-COOH, bTiO_2_-Gd-GEM, bTiO_2_-Gd-IGF1, bTiO_2_-Gd-IGF1-GEM, and irradiated by 1.2 W cm^−2^ of 808 nm NIR, while bTiO_2_-Gd-IGF1-GEM nanoprobes induce more extensive cell death. The live/dead cell staining results are consistent with the MTT results, further indicating that the photothermal-chemotherapy has a more significant killing effect, and can overcome drug-resistance of MIA PaCa-2 PDAC cells.

### Photothermal imaging, MRI, and synergetic photothermal-chemotherapy in vivo

The in vivo toxicity assessment of the nanoprobe is carried out by injecting PBS, bTiO_2_-Gd-COOH, bTiO_2_-Gd-IGF1 into balb/c mice through tail vein. Additional file 1: Fig. S4a shows the weight change of mice within 1 month after nanoprobe injection. Additional file 1: Fig. S4b, c are blood routine index and the histological analysis of the organs after injection for 1 month. Relative to PBS controls, we observed no marked changes in body weight and blood routine indexes, meanwhile no damage in tissues or other lesions like pulmonary fibrosis, inflammatory, or necrosis are evident in bTiO_2_-Gd-COOH and bTiO_2_-Gd-IGF1 administered groups. The above results indicate that the prepared nanoprobes offer no toxicity to mice at the doses used for 1 month.

**Fig. 7:**
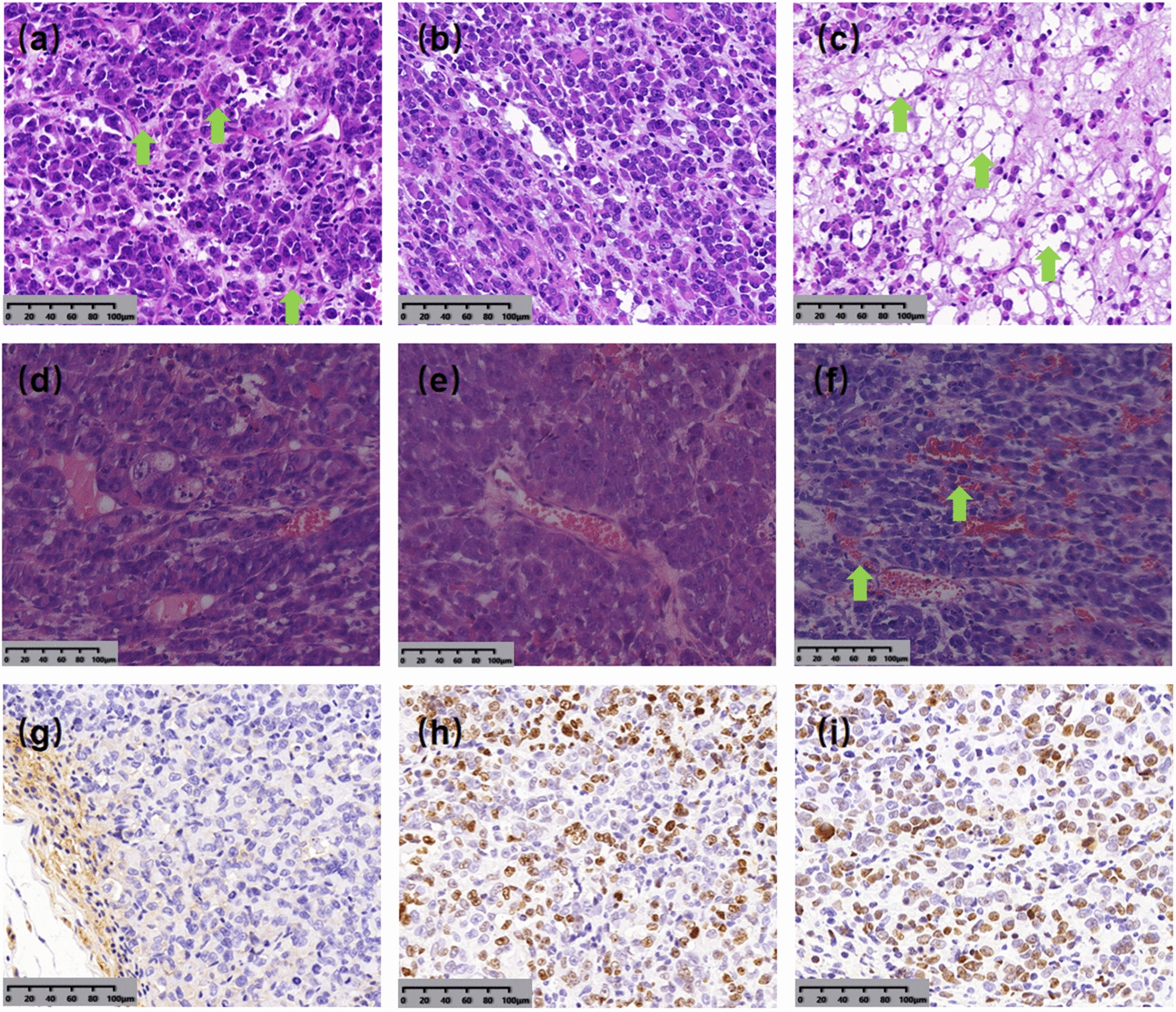
**a**–**c** H&E staining of fibrous matrix in tumor and fibrous matrix loosening under photothermal process; H&E staining of vascular exudation in tumor for treatment of PBS (**d**), bTiO_2_-Gd-IGF1 (**e**) and bTiO_2_-Gd-IGF1 + NIR (**f**). **g** Immunohistochemical results of IGF1 receptor expression. Ki67 immunohistochemical results of in PBS (**h**) and bTiO_2_-Gd-IGF1 treatment (**i**). Scale bar is 100 μm

After the mouse pancreatic cancer tumor model was constructed, HE staining was performed on tumor tissue. In Fig. 7a, the arrows show that tumor contained a large amount of stroma cells, which suggests the tumor model is successfully constructed. In order to detect the matrix barrier loosening and vascular permeability improving in photothermal process, bTiO_2_-Gd-IGF1 nanoprobes were injected into the tumor site, and then underwent irradiation with or without 808 nm NIR laser. It can be seen from Fig. 7b–f, compared with unirradiated control group (Fig. 7a), the tumor matrix is loosened, the vascular permeability is increased, and the red blood cells extravasate into the surrounding tissue after near-infrared irradiation. Immunohistochemical analysis (Fig. 7g) shows that IGF1R is ubiquitously found in both of tumor cells and fibrous stromal cells, which is consistent with literature reports [6, 27]. Subsequently, we examined the impact of bTiO_2_-Gd-IGF1 on the proliferation of PDAC tumor cells in vivo. Studies have found that IGF1 polypeptide can promote tumor cell proliferation, while Zhou et al. demonstrated IGF1 modified iron oxide nanoparticles could reduce IGF1’s growth-stimulating function, but retain the targeting ability [6]. To further confirm this phenomenon, we injected the tumor with PBS or bTiO_2_-Gd-IGF1 twice, and then analyzed with Ki67 immunohistochemistry. As depicted in Fig. 7h, i, relative to PBS controls, repeatedly injected tumor with bTiO_2_-Gd-IGF1 does not enhance cell proliferation index. Therefore, modified bTiO_2_-Gd-COOH with IGF1 could decrease IGF1’s growth-stimulating activity but still retain targeting ability. Nevertheless, its underlying mechanism is still undetermined.

**Fig. 8:**
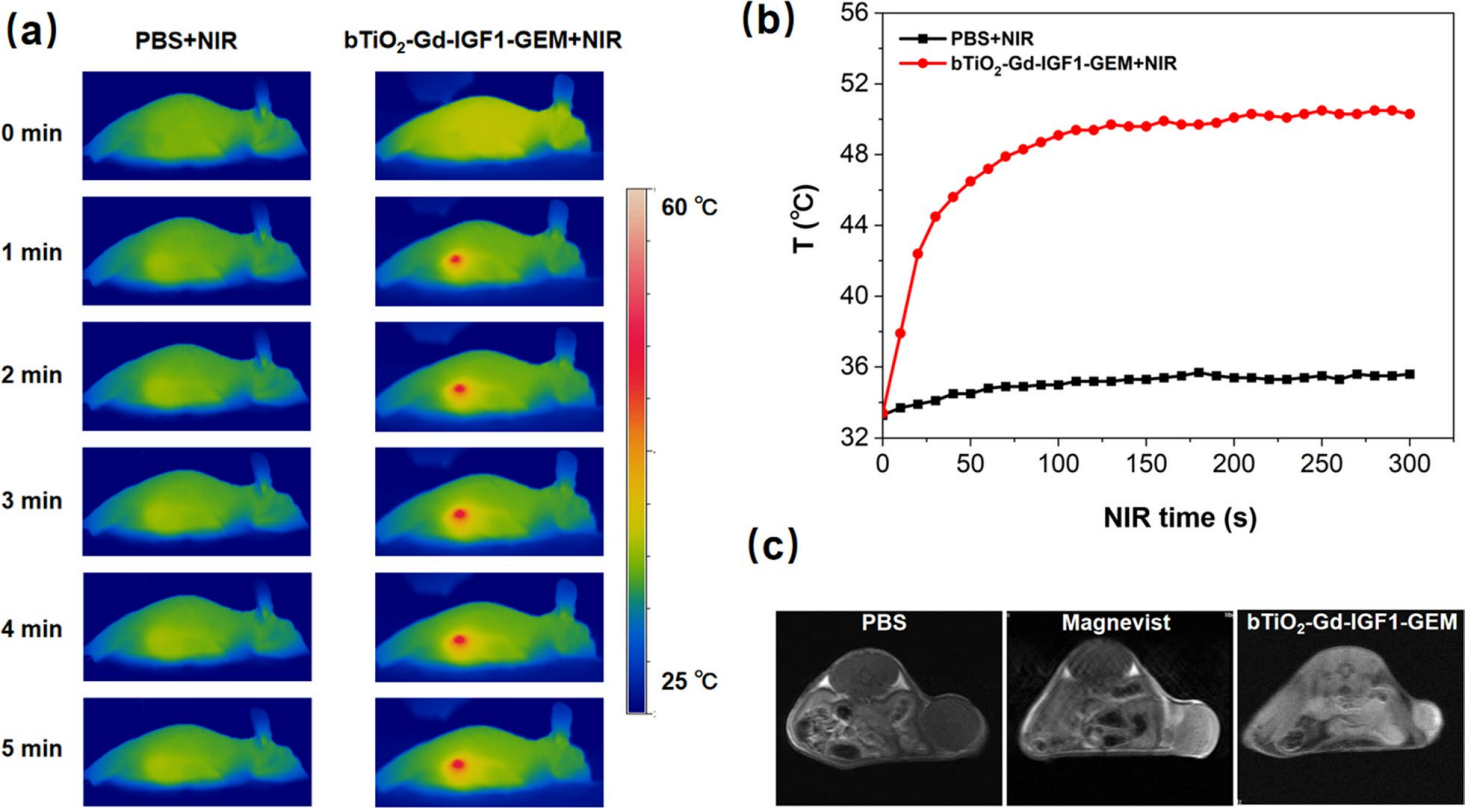
**a** Photothermal illustrations of tumor in the PBS and bTiO_2_-Gd-IGF1-GEM groups receiving 5 min irradiation of NIR at 1.2 W cm^−2^. **b** Tumor temperature alterations during the irradiation of NIR in **a**. **c** MRI tumor images from PBS group, Magneist group and bTiO_2_-Gd-IGF1-GEM group

Followed, photothermal images of tumors are analyzed in PBS group and bTiO_2_-Gd-IGF1-GEM group under NIR irradiation. As depicted in Fig. 8a, relative to controls, tumoral temperature rapidly increase in NIR irradiated bTiO_2_-Gd-IGF1-GEM group. In Fig. 8b, the tumoral temperature in PBS group increases from 33.3 to 35.6 °C after NIR irradiation, indicating that NIR is safety. While tumoral temperature in the bTiO_2_-Gd-IGF1-GEM group rises from 33.4 to 50.3 °C after NIR irradiation, cancer cells can be rapidly eliminated after only a few minutes of exposure. Hence, the bTiO_2_-Gd-IGF1-GEM group after NIR irradiation can easily kill cancer cells according to previous study suggested expose of cells in 50 °C [41]. Figure 8c shows MRI images in various groups. As shown in the picture, the intratumoral T_1_-weighted MR imaging of PBS is poor, while bTiO_2_-Gd-IGF1-GEM nanoprobe is better than that of Magnevist at the same Gd concentration, which confirms the feasibility MRI performance of bTiO_2_-Gd-IGF1-GEM nanoprobes.

**Fig. 9:**
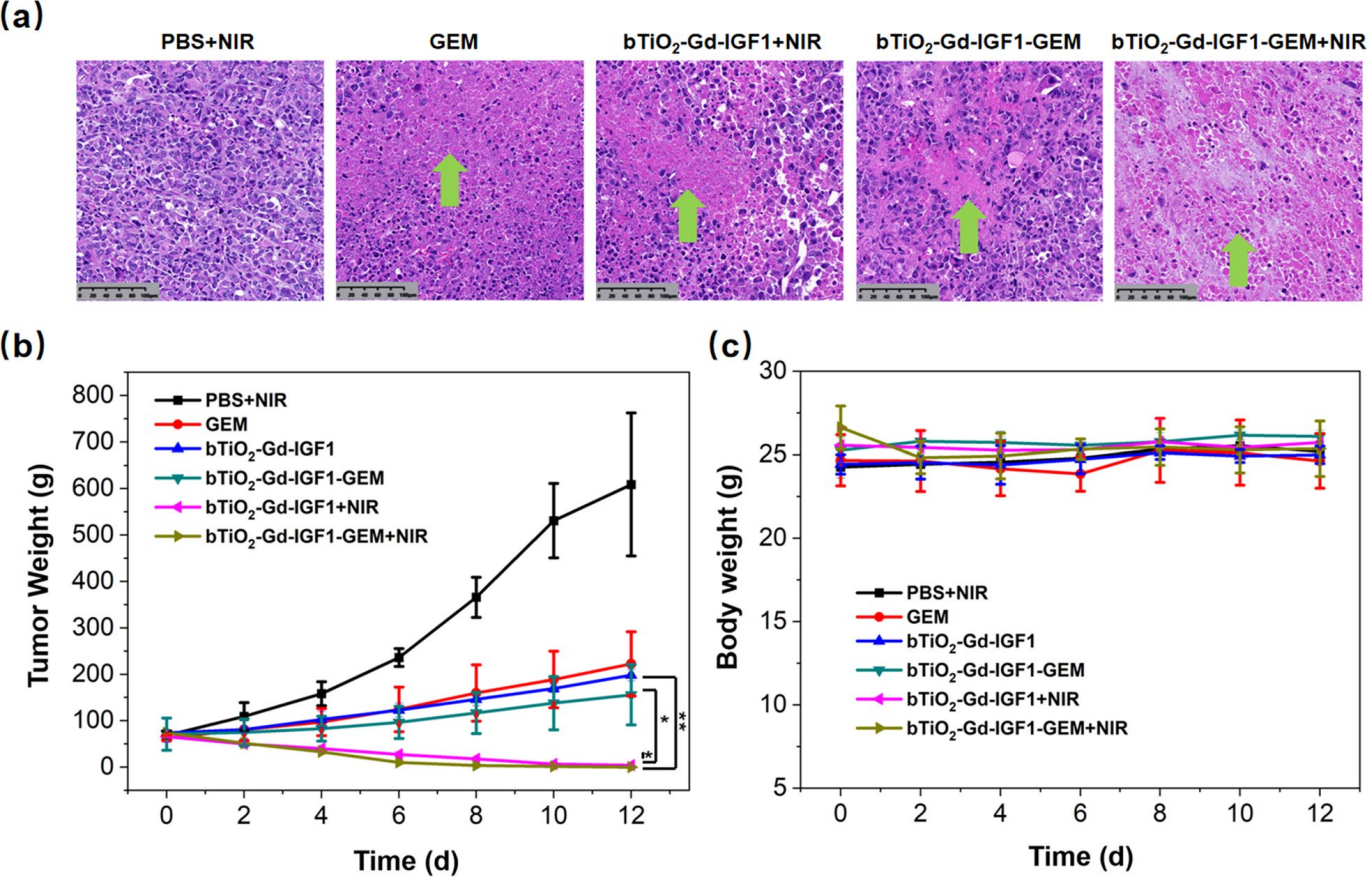
**a** Tumor injury analysis via H&E staining following injection of mice with PBS, GEM, bTiO_2_-Gd-IGF1 and bTiO_2_-Gd-IGF1-GEM, followed by irradiation, in the presence or absence of for 5 min. **b** Relative tumor volume and **c** Mice body weights following aforementioned treatments. Data are presented as mean ± standard deviation (n = 3). *p < 0.05, **p < 0.01 were evaluated via Student’s t test, and used as the significance threshold

To evaluate in vivo synergetic photothermal-chemotherapy, the tumor-injected animals were arbitrarily separated into six classes. The PBS + NIR group was PBS administered, followed by irradiation with NIR laser. The GEM group was administered with GEM. The bTiO_2_-Gd-IGF1 + NIR group was bTiO_2_-Gd-IGF1 administered, followed by irradiation with NIR. Likewise, the bTiO_2_-Gd-IGF1-GEM or bTiO_2_-Gd-IGF1-GEM + NIR group was bTiO_2_-Gd-IGF1-GEM administered, followed by irradiation without or with NIR, respectively. In order to prove that cooperative photothermal-chemotherapy can loosen matrix barrier and overcome drug-resistance, part of mice were euthanized shortly following the irradiation of NIR, and tumors were assessed via H&E staining. According to Fig. 9a, no marked pathological damage was observed in the PBS + NIR group. There are different degrees of cell damage in the GEM, bTiO_2_-Gd-IGF1 and bTiO_2_-Gd-IGF1-GEM groups, the green arrows in the figure represent necrosis of tumor tissue. In the bTiO_2_-Gd-IGF1-GEM + NIR group, most of the cancer cells are eliminate, indicating that advantages of synergetic therapy. Figure 9b provides the changes of tumor volume after treatments, based on the treatments in Fig. 9a. Following 12 days, the tumor sizes are 609.0 ± 153.7, 222.5 ± 69.3, 198.2 ± 12.6, 156.0 ± 64.9, 4.1 ± 1.3 and 0.3 ± 0.4 mm^3^ in the PBS + NIR, GEM, bTiO_2_-Gd-IGF1, bTiO_2_-Gd-IGF1-GEM, bTiO_2_-Gd-IGF1 + NIR and bTiO_2_-Gd-IGF1-GEM + NIR groups, respectively. Relative to the bTiO_2_-Gd-IGF1 + NIR or bTiO_2_-Gd-IGF1-GEM group, the tumors in bTiO_2_-Gd-IGF1-GEM + NIR group significantly reduce, which indicate synergetic photothermal-chemotherapy not only can loosen matrix barrier, but also can overcome drug-resistance. The toxic effects of the treatments were evaluated by measuring body weight of the mice within 12 days. In Fig. 9c, body weights of mice in each group did not change significantly, which proves that the treatments are safety and tolerable in pancreatic tumor models.

## Conclusions

In summary, to break matrix barrier and reverse drug resistance in PDAC, this study proposes a dual-targeted photothermal-chemotherapy strategy in PDAC cells and stromal cells, and prepare bTiO_2_-Gd-GEM-IGF1 nanoprobes. First, WB and pathology results confirm IGF1R is ubiquitously found in MIA PaCa-2 cells and their tumor model, meanwhile MIA PaCa-2 cells are resistant to gemcitabine. The IGF1 modified nanoprobes exhibit stronger targeting ability to MIA PaCa-2 cells. Next, cell experiments show that the killing effect of synergistic treatment on MIA PaCa-2-resistant cells is about 3.3 times than chemotherapy alone, which suggest reversal of drug-resistance. Followed, histological experiments prove photothermal can loosen the fibrous matrix of pancreatic cancer model and increase the permeability of blood vessels. Therefore, in vivo results show that tumor models are almost completely cured after 12 days of combined treatments. In conclusion, we provide a more effective approach for treating pancreatic cancer through matrix barrier loosening and drug resistance reverse, and also provide an experimental basis for applying black TiO_2_ in synergistic photothermal-chemotherapy in pancreatic cancer.

## Supplementary Information

**Additional file 1: Figure S1.** PH and NIR co-responded drug-release of bTiO_2_-Gd-IGF1-GEM. **Figure S2.** The IGF1R expression in MIA PaCa-2 cells, hTERT-HPNE cells, BxPC-3 cells, and Panc-2 cells. **Figure S3.** Live/dead cell staining following various treatments. Green and red fluorescence denote live and dead cells, respectively. Scale bar is 20 μm. **Figure S4.** In vivo toxicity analysis of nanoprobes on balb/c mice. The changes of (a) body weight, (b) routine blood indexes, and (c) organ histological analysis after injected with PBS, bTiO_2_-Gd-COOH, bTiO_2_-Gd-IGF1 for 1 month. Scale bar is 100 μm.

## Data Availability

All the data generated or analyzed during this study are included in the article.
