# Supplementary material for: IGF1 receptor-targeted black TiO2 nanoprobes for MRI-guided synergetic photothermal-chemotherapy in drug resistant pancreatic tumor
Source: J Nanobiotechnology. 2022 Jul 6;20:315. doi: 10.1186/s12951-022-01525-3 (PMC9258211; doi:10.1186/s12951-022-01525-3)
Supplement: Supplementary file 1 — Additional file 1: Figure S1. PH and NIR co-responded drug-release of bTiO2-Gd-IGF1-GEM. Figure S2. The IGF1R expression in MIA PaCa-2 cells, hTERT-HPNE cells, BxPC-3 cells, and Panc-2 cells. Figure S3. Live/dead cell staining following various treatments. Green and red fluorescence denote live and dead cells, respectively. Scale bar is 20 μm. Figure S4. In vivo toxicity analysis of nanoprobes on balb/c mice. The changes of (a) body weight, (b) routine blood indexes, and (c) organ histological analysis after injected with PBS, bTiO2-Gd-COOH, bTiO2-Gd-IGF1 for 1 month. Scale bar is 100 μm. [file 12951_2022_1525_MOESM1_ESM.doc]

**Additional file 1**

**IGF1 receptor-targeted** **black TiO_2_ nanoprobes for MRI-guided synergetic photothermal-chemotherapy in drug resistant pancreatic tumor**

Kaiwei Xu^1#^, Lufei Jin^1#^, Liu Xu^1#^, Yuchao Zhu^1^, Lu Hong^1^, Chunshu Pan^2^, Yanying Li^2, 3^, Junlie Yao^2, 3^, Ruifen Zou^2, 3^, Weiwei Tang^1^, Jianhua Wang^1*^, Aiguo Wu^2,3*^, Wenzhi Ren^2, 3, 4*^

1. Department of Radiology, the Affiliated Hospital of Medical School, Ningbo University, 247 Renmin Road, Jiangbei District, Ningbo, Zhejiang Province, 315020, China

2. Cixi Institute of Biomedical Engineering, International Cooperation Base of Biomedical Materials Technology and Application, Chinese Academy of Science (CAS) Key Laboratory of Magnetic Materials and Devices and Zhejiang Engineering Research Center for Biomedical Materials, Ningbo Institute of Materials Technology and Engineering, CAS, Ningbo 315201, P.R. China

3. Advanced Energy Science and Technology Guangdong Laboratory, Huizhou 516000, P.R. China

4. Key Laboratory of Diagnosis and Treatment of Digestive System Tumors of Zhejiang Province, Ningbo 315016, China

^#^Kaiwei Xu, Lufei Jin and Liu Xu contributed equally to this work.

^*^Corresponding author, E-mail: wangjianhua@nbu.edu.cn, aiguo@nimte.ac.cn, renwzh@nimte.ac.cn


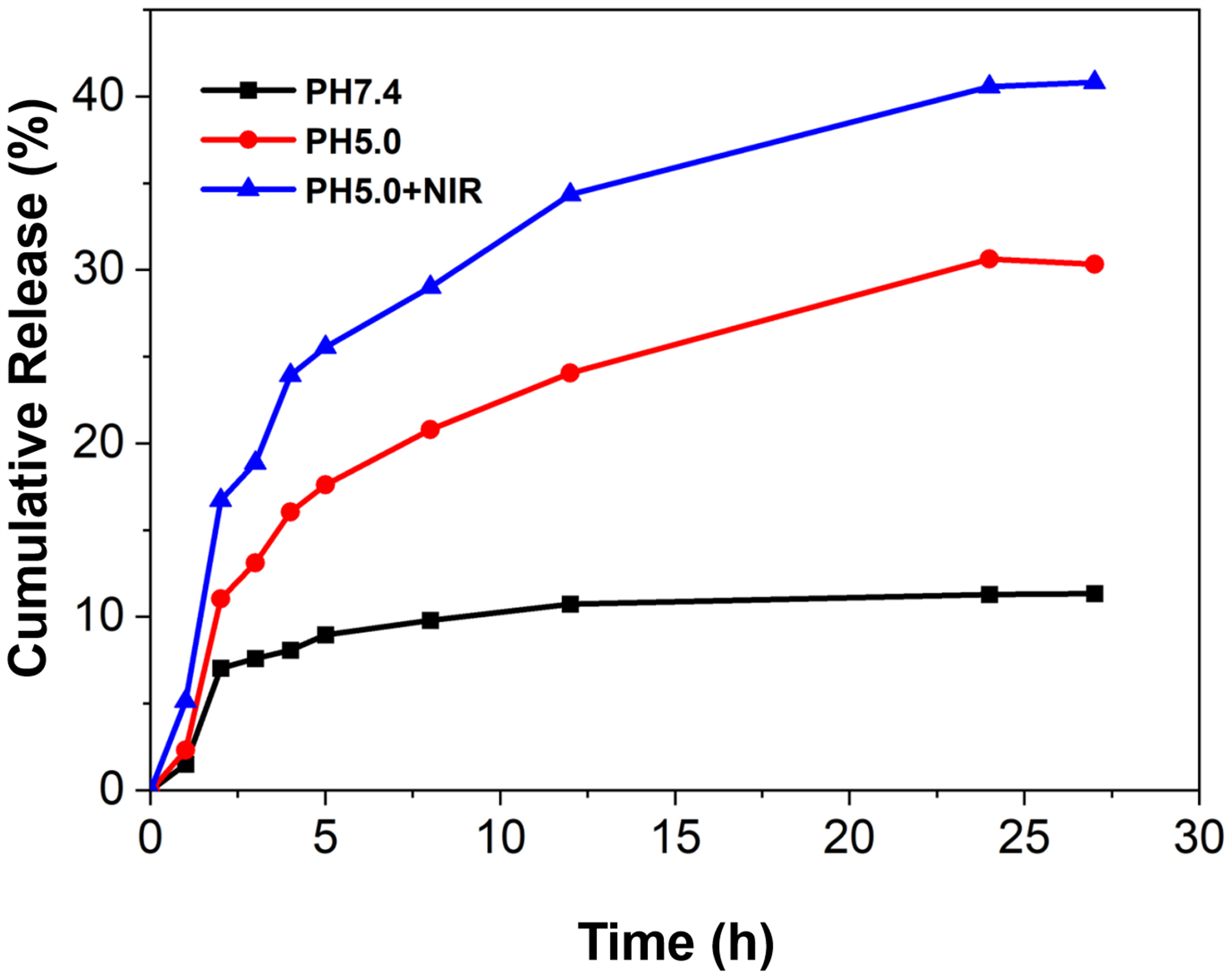


**Figure S1.** PH and NIR co-responded drug-release of bTiO_2_-Gd-IGF1-GEM.


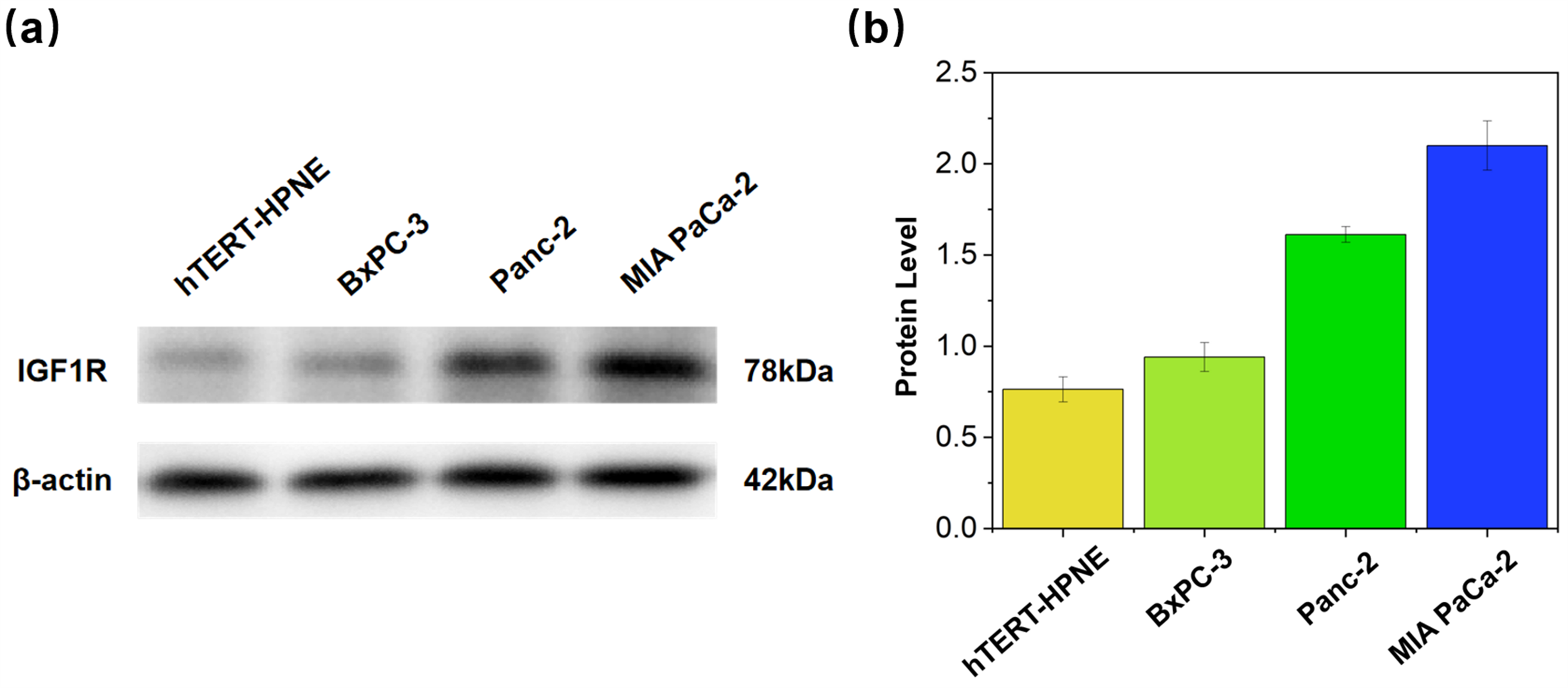


**Figure** **S2.** The IGF1R expression in MIA PaCa-2 cells, hTERT-HPNE cells, BxPC-3 cells, and Panc-2 cells.


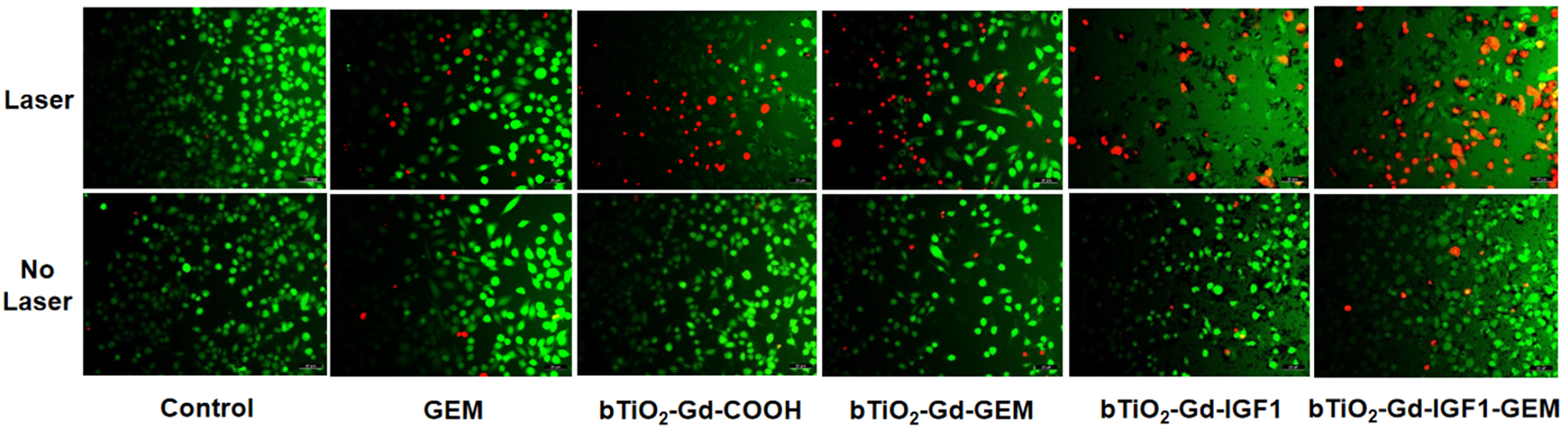


**Figure** **S3.** Live/dead cell staining following various treatments. Green and red fluorescence denote live and dead cells, respectively. Scale bar is 20 μm.


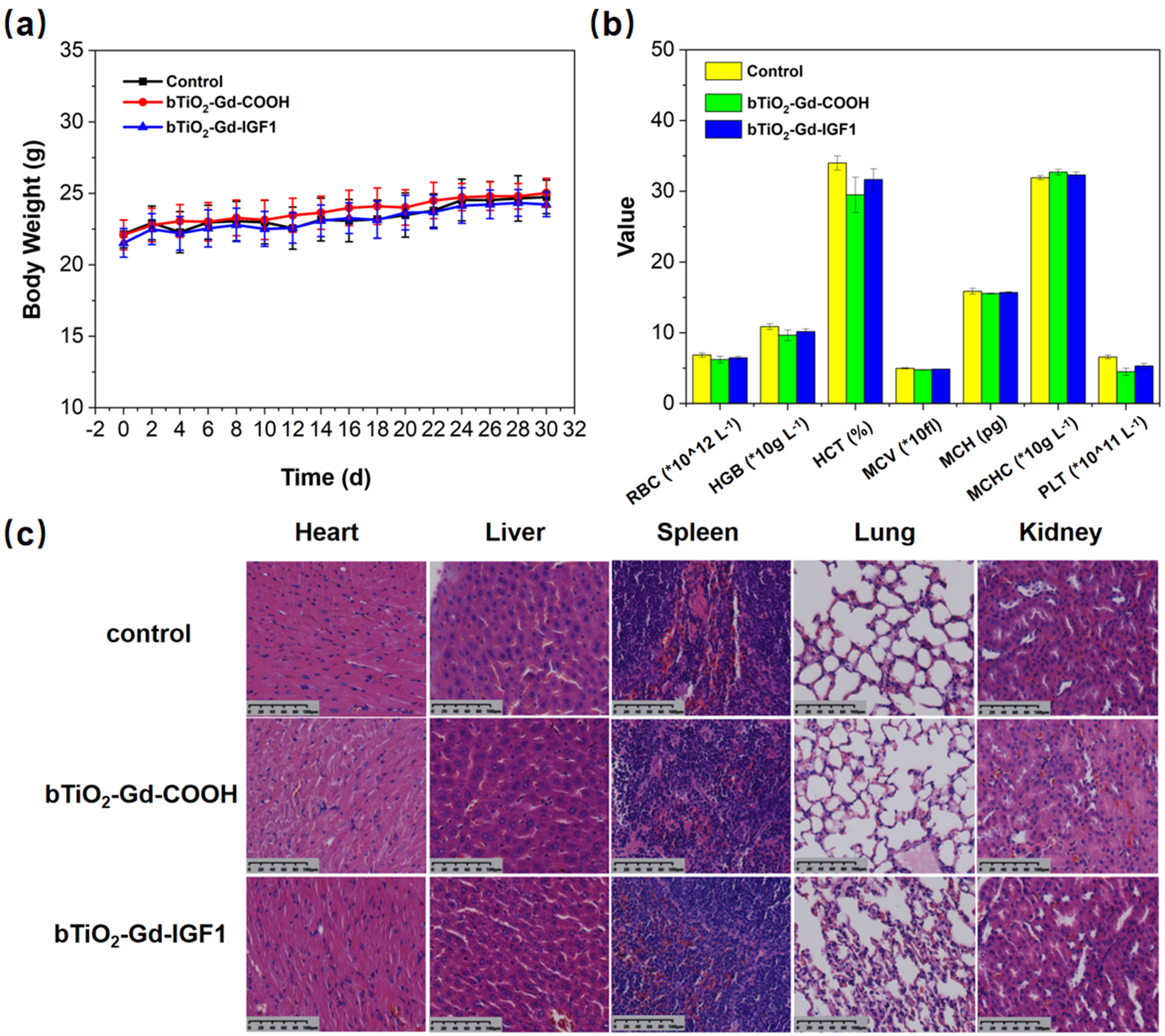


**Figure S4.** In vivo toxicity analysis of nanoprobes on balb/c mice. The changes of (a) body weight, (b) routine blood indexes, and (c) organ histological analysis after injected with PBS, bTiO_2_-Gd-COOH, bTiO_2_-Gd-IGF1 for one month. Scale bar is 100 μm.
